# C1494T mitochondrial dna mutation, hearing loss, and aminoglycosides antibiotics

**DOI:** 10.1016/S1808-8694(15)30554-1

**Published:** 2015-10-19

**Authors:** Mariana Postal, Bruna Palodeto, Edi Lúcia Sartorato, Camila Andréa de Oliveira

**Affiliations:** 1 4th year student of biomedicine, Hermínio Ometto University - UNIARARAS; 2 24th year student of biomedicine, Hermínio Ometto University - UNIARARAS; 3 Associate Professor, Researcher - Center of Molecular Biology and Genetic Engineering - CBMEG-UNICAMP; 4 PhD. Assistant Professor - Health Sciences Nucleus - NUCISA - Hermínio Ometto University - UNIARARAS

**Keywords:** aminoglycosides, dna, mitochondrial, mutation

## Abstract

In view of the complex mechanism of hearing, it is not difficult to understand that hearing impairment may result from a wide variety of genetically determined anomalies and various environmental factors. Specific mutations in the mitochondrial DNA 12S rRNA gene are responsible for maternally inherited non-syndromic hearing loss, and for increased susceptibility to the ototoxicity of aminoglycoside antibiotics.

**Aim:**

To asses the presence of C1494T mutation among individuals with normal hearing and hearing impairment who used aminoglycosides and those who had not had contact with the antibiotic.

**Material and Method:**

The study was composed of 20 patients with ***nonsyndromic sensorineural hearing loss without prior use of aminoglycosides and 40 premature and high-risk newborns who used ototoxic drugs, of whom 20 had good hearing and 20 had hearing loss. The samples were analyzed by PCR-RFLP with the restriction enzyme Hph I.

**Study Design:**

Experimental.

**Results:**

The mitochondrial 12S rRNA C1494T mutation was not detected in any of the samples analyzed.

**Conclusion:**

Our data suggest that the hearing loss of the individuals we analyzed was not related to the ototoxicity of mutation C1494T, showing that this mutation is not frequent in our population.

## INTRODUCTION

In recent years, there has been a major progress in our understanding of hearing mechanisms; numerous genes involved in this process have been discovered and the ways by which they interact has been broadly investigated. The hearing deficiency etiology can involve mutations in one single gene or a combination of mutations in different genes, and also environmental factors, including perinatal infection, brain trauma involving the cochlea or ototoxic drugs -aminoglycosides, or even the interaction between environmental and genetic factors^1^.

Mutations in mitochondrial DNA (mtDNA) are responsible for a variety of disorders which affect numerous organs and tissues^2^. Many of the mtDNA mutations described in the literature are associated with syndromic and non-syndromic forms of hearing impairment^3^,^4^. The mitochondria are responsible for the availability of power to the cells in the form of adenosine triphosphate (ATP). Thus, the organs which require more energy are the ones that present functional alterations in cases of mitochondrial DNA mutations, such as nerve, muscle, endocrine, optical and auditory cells. As the cochlea consumes large amounts of energy, alterations in the mitochondrial DNA in the hair cells cause hearing impairment in a ratio of 0.5% to 1% of all the genetic hearing impairment^5^.

Most molecular alterations found in the mitochondrial DNA, which are associated with hearing impairment are present in genes 12S rRNA and tRNA. Especially, mutations affecting gene 12S rRNA are responsible for non-syndromic sensorineural hearing loss and a raise in susceptibility to the ototoxicity caused by aminoglycoside antibiotics^6^,^7^.

These antibiotic agents are clinically important drugs, used everywhere in the world, especially in developed countries, in order to control bacterial infections in hospitalized patients. Notwithstanding, in developing countries these drugs are still routinely used in lesser infections^8^. Aminoglycoside antibiotic agents act by binding directly to the subunit 16S and 30S of bacterial ribosomal RNA (rRNA), causing a premature termination of the protein synthesis. The frequent use of these drugs causes toxicity involving the renal, vestibular and auditory systems. The renal damage is usually reversible, but the auditory and vestibular ototoxicity is frequently irreversible^9^.

Literature reports that aminoglycoside ototoxicity may be associated with a genetic predisposition with a dominant autosomal inheritance pattern, recessive, X-linked or mitochondrial. In family cases of ototoxic hearing impairment caused by aminoglycoside hypersensitivity is frequently transmitted by maternal inheritance, suggesting involvement of mitochondrial genome^7^.

Studies held with the mitochondrial genome with patients with aminoglycoside ototoxicity revealed the identification of mitochondrial DNA mutations in gene 12S rRNA. A1555G mutation was the first mitochondrial mutation described^10^, and since then it has been identified in many families, of different ethnics, segregating with non-syndromic hearing impairment with or without exposure to aminoglycosides^11^. A little while ago, a C → T transition in position 1494 of gene 12S rRNA was identified in a Chinese family with non-syndromic sensorineural hearing impairment, including individuals affected with and without prior exposure to aminoglycosides^12^ and, subsequently, in other two Chinese families^13^ and three Spanish ones^14^. The clinical heterogeneity of hearing impairment in these cases is directly related to the age of administration onset and with the duration of drug exposure. The younger the age of administration onset, the greater the severity of the hearing impairment developed by the patient^15^.

The A1555G mutation is structurally equivalent to mutation C1494T. They are both located in a highly preserved region of the 12S rRNA, associated with the binding of the aminoglycoside to the bacteria^11^. These mutations seem to alter the secondary structure of the 12S rRNA, in such a way as to be similar to the bacterial ribosomal subunit, thus leading to an increase in the susceptibility of aminoglycosides and consequently its ototoxic effect^10^.

Many biochemical studies report the pathogenic potential of mutation C1494T, indicating that aminoglycosides modulate the hearing impairment expression potential and penetrance when associated with such mutation^13^. Nonetheless, mutation C1494T was not described in any family of other races so far^16^,^17^. In this study, we investigated the presence of mitochondrial mutation C1494T among the patients with hearing impairment and with normal hearing exposed to aminoglycosides and the patients with hearing impairment who did not have contact with the antibiotic.

## MATERIALS AND METHODS

### Series

This study was characterized by a clinical and experimental research (Ethics Committee Approval # 484/2006), of individuals with hearing impairment without exposure to aminoglycoside antibiotics and in normal-hearing and hearing impaired newborns, premature and of high risk who remained in the NICU (neonatal intensive care unit) and had contact with the antibiotic.

The series was made up of 60 individuals, 20 with hearing impairment and no history of sensitization regarding the use of aminoglycosides (Group B), 20 hearing individuals (Group C) and with audiologic diagnosis of non-syndromic sensorineural hearing loss (Group A, n=20) who used aminoglycosides.

### Gene DNA extraction

Sample analysis was carried out in DNA isolated by the phenol-chloroform method^18^ from peripheral blood leucocytes after obtaining the patients' free and informed consent.

### 12S rRNA Gene Amplification

The individuals were previously analyzed as to the presence of mutations to genes GJB2 and GJB6, since mutations to these genes are responsible for a large proportion of cases of non-syndromic hearing loss with recessive autosomal inheritance^19^,^20^, and A1555G mitochondrial mutations to gene 12S rRNA^21^ and A7455G at gene tRNA(SerUCN)^22^. Among the individuals who did not have such mutations we investigated mutation C1494T.

From the samples of DNA extracted from the peripheral blood, a 441pb fragment from the 12S rRNA gene sequence (GenBank: NC_001807), was amplified by PCR using primers C1494TF (5′-GTCGAAGGTGGATTTAGCAGT-3′) and C1494TR (5′-GCAGAAGGTATAGGGGTTAG-3′). The amplification reaction was carried out using 200 to 500ng of genomic DNA, 10mM of solution containing deoxynucleotides (dATP, dCTP, dGTP and dTTP), 10pmol/ml of each primer; 2,0U of Taq DNA polymerase; PCR 10X buffer (Tris-HCl 10mM pH 8.8) and 3mM of MgCl2, in a final volume of 50ml, under the following conditions: a denaturation cycle at 95°C for 5 min; 30 denaturation cycles at 94°C for 30s; annealing at 54°C for 2 min, extension at 65°C for 1mim; 7 min of final extension at 65°C. Amplification products were submitted to electrophoresis in 1% agarose gel and subsequently dyed in ethide bromide.

C1494T mutation identification on gene 12S rRNA

Molecular investigation of mutation C1494T on the 12S rRNA gene was carried out by analysis of the polymorphism of the restriction fragments (PCR-RFLP) using enzyme Hph I, according to manufacturer's specifications and, analyzed in 1.5% agarose gel. Allele 1494T abolishes the Hph I site in the sequence creating a fragment of 441pb. Thus, individuals with the 1494C allele have fragments of 370pb and 71pb.

## RESULTS

**Figure 1:**
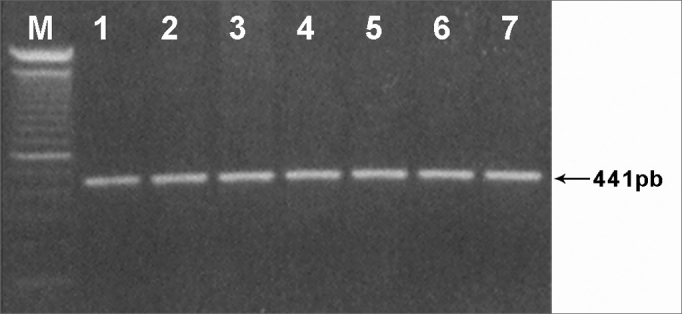
441pb fragment from gene 12S rRNA amplified by PCR and analyzed in 1% agarose gel (M) - molecular weight marker (100pb).

**Figure 2:**
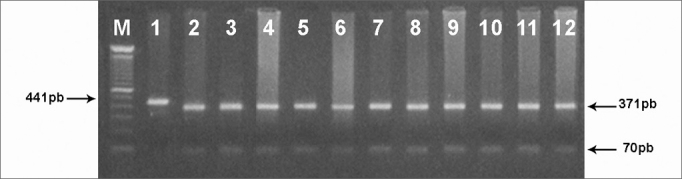
Tracking C1494T mitochondrial mutation by PCR-RFLP. A fragment of 441pb was cleaved with the Hph I restriction enzyme. Normal mitochondrial DNA generates wto restriction fragments (370pb and 71pb) and the mutant loses the restriction site and not' cleaved by the enzyme (441pb). (M) - molecular weight marker (100pb); (1)-undigested PCR product. (2-12) - patients without the C1494T mutation.

In an attempt to clear up the molecular aspects of the aminoglycosides' ototoxic effects, we tracked down the C1494T mutation in 40 patients with non-syndromic sensorineural hearing loss. Of these, 20 patients had a history of using aminoglycosides. Other 20 normal hearing individuals exposed to aminoglycosides, were also studied. After amplifying a fragment of gene 12S rRNA (441pb) (Figure 1) and subsequent Hph I digestion (Figure 2) of the 60 DNA samples of patients from groups A, B and C, we did not identify mutation C1494T in any of the samples analyzed.

## DISCUSSION

Functional studies show that the C1494T mutation causes a mild mitochondrial dysfunction and sensitivity to aminoglycosides, therefore indicating that the C1494T mutation alone is not responsible for the hearing impairment phenotype. Nonetheless, the hearing loss can be increased by external modifying factors, such as aminoglycosides, and gene interactions. In cases when there is no exposure to aminoglycosides, the C àT transition in position 1494 of gene 12S rRNA has, in vivo, a weak pathogenic potential^15^.

Aminoglycoside ototoxicity is due to their binding to the receptors of inner ear hair cells. The aminoglycoside-receptor complex formation causes changes to the cell membrane, to its permeability, affecting cilia function and, later causing the destruction of these cells^23^. In the presence of mutation C1494T the hearing cells become more susceptible, because the base change in a highly preserved region of the 12S rRNA, makes the region similar to the bacterial ribosomal subunit^11^.

Data reported from a Chinese family show that the interaction of mutation C1494T with other genes, in which those individuals with this mutation had hearing impairment regardless of exposure to aminoglycosides. On the other hand, it has also been disclosed that individuals with the C1494T mutation who were not exposed to the antibiotic did not have hearing loss^12^.

In the present study, we assessed the presence of C1494T mutation in hearing individuals and those with hearing impairment who used aminoglycosides and those who did not use the antibiotic. Nonetheless, we did not find positive cases for this mutation, reinforcing the results found in previous studies, who reported that the C1494T mutation is not very frequent in the world population^13^,^16^,^17^,^24^.

## CONCLUSION

Our data suggests that the hearing impairment in the analyzed individuals is not related to the ototoxicity of mutation C1494T. Nonetheless, the lack of C1494T mutation in the mitochondrial genome of these individuals does not rule out the possibility of existing mutations in other genes not evaluated in this study. Thus being, later studies with other genes are necessary to clarify the hearing impairment etiology of these individuals.
